# Supplementary material for: rRNA methylation by Spb1 regulates the GTPase activity of Nog2 during 60S ribosomal subunit assembly
Source: Nat Commun. 2023 Mar 2;14:1207. doi: 10.1038/s41467-023-36867-5 (PMC9981671; doi:10.1038/s41467-023-36867-5)
Supplement: Supplementary file 1 — Supplementary Information [file 41467_2023_36867_MOESM1_ESM.pdf]

Supplementary Information

rRNA methylation by Spb1 regulates the GTPase activity of Nog2 during 60S ribosomal subunit assembly

Kamil Sekulski†, Victor Emmanuel Cruz†, Christine S. Weirich and Jan P. Erzberger[1]*

Department of Biophysics, UT Southwestern Medical Center; 5323 Harry Hines Blvd., ND10.104B, Dallas, TX 75390-8816, USA

*Corresponding author. Email: jan.erzberger@utsouthwestern.edu

† These authors contributed equally

Supplementary Information Table 1 | Cryo-EM data collection, refinement and validation statistics

Supplementary Information Table 2 | Yeast strains used in this study

Supplementary Information Table 3 | Plasmids used in this study

Supplementary Information Figure 1 | Purified Nog2·Tif6 pre-ribosomes used for cryo-EM analysis.

Supplementary Information Figure 2 | Micrographs, 2D classes, Fourier shell correlation (FSC) curves and ResMap plots of maps from *SPB1* (panels a-e) or *spb1*$^{D52A/E769K}$ (panels f-h) strains.

Supplementary Information Figure 3 | Micrographs, 2D classes, Fourier shell correlation (FSC) curves and ResMap plots of maps of Nog2^{pre} and Nog2^{post} (panels a-e) and Nog2^{pre}+ AlF_4- (panels f-h) purified from the *spb1*^{D52A} strain.

Supplementary Information Figure 4 | Cryo-EM data processing, 3D-classification and particle sorting scheme.

Supplementary Information Figure 5 | Representative Cryo-EM map densities for the wild-type Nog2^{pre} sample.

Supplementary Information Figure 6 | Map interpretation.

Supplementary Information Figure 7 | Cryo-EM data processing, 3D-classification and particle sorting scheme for Nog2·Tif6 intermediates.

Supplementary Information Figure 8 | Overexpression of *NMD3* or *SPB1-MTD* does not rescue growth of *spb1*^{D52A} and location of *spb1*^{D52A} suppressor mutants in the C-terminal domain of Spb1.

Supplementary Information Table 1 | Cryo-EM data collection, refinement and validation statistics

	Nog2^{pre}<br>*SPB1*<br>(emdb-26651)<br>(pdb 7UOO)	Nog2^{pre}<br>5S rRNP<br>*SPB1*<br>(emdb-26689)	Nog2^{pre}<br>*spb1*^{D52A}<br>(emdb-26703)<br>(pdb 7UQZ)	Nog2^{post}<br>*spb1*^{D52A}<br>(emdb-26799)<br>(pdb 7UUI)	Nog2^{pre}-AlF_4^-<br>*spb1*^{D52A}<br>(emdb-26686)<br>(pdb 7UQB)	Nog2^{pre}<br>*spb1*$^{D52A/E769E}$<br>(emdb-26941)<br>(pdb 7V08)
**Data collection and processing**						
Magnification	81,000x	81,000x	81,000x	81,000x	81,000x	81,000x
Voltage (kV)	300	300	300	300	300	300
Electron exposure (e^-/Å^2)	56	56	56	56	50	56
Defocus range (μm)	-0.9 – (-2.2)	-0.9 – (-2.2)	-0.9 – (-2.2)	-0.9 – (-2.2)	-0.9 – (-2.2)	-0.9 – (-2.2)
Pixel size (Å)	1.08	1.08	1.08	1.08	1.07	1.08
Symmetry imposed	C1	C1	C1	C1	C1	C1
Initial particle images (no.)	1,639,317	1,639,317	905,023	905,023	716,018	1,207,583
Final particle images (no.)	1,159,206	1,035,794	328,470	50,091	417,994	320,145
Map resolution (Å)	2.34	2.63	2.44	2.90	2.38	2.36
FSC threshold	0.143	0.143	0.143	0.143	0.143	0.143
Map resolution range (Å)	2.2 – 10	2.2 – 10	2.2 – 10	2.2 – 10	2.2 – 10	2.2 – 10
**Refinement**						
Initial model used (PDB code)	3JCT		3JCT	6YLH	3JCT	3JCT
Model resolution (Å)	2.52		2.60	3.33	2.53	2.48
FSC threshold	0.5		0.5	0.5	0.5	0.5
Map sharpening *B* factor (Å^2)	79.52		82.93	87.17	70.61	61.93
Model composition						
Non-hydrogen atoms	157949		157839	3672	157949	157849
Protein residues						
Nucleotides	10806		10806	452	10806	10806
	3329		3325	1	3330	3325
B factors (Å^2)						
Protein	22.13		19.42	21.70	19.17	21.39
Nucleotides	23.34		21.14	17.29	20.90	32.00
Ligand	15.98		11.19	12.13	12.04	12.91
Waters	16.23		11.01		10.89	8.39
R.m.s. deviations						
Bond lengths (Å)	0.002		0.002	0.002	0.003	0.005
Bond angles (°)	0.563		0.536	0.504	0.576	0.729
Validation						
MolProbity score	1.31		1.23	1.13	1.28	1.29
Clashscore	3.41		3.67	3.38	4.24	4.08
Poor rotamers (%)	0.2		0.33	0	0.43	0.55
Ramachandran plot						
Favored (%)	97.56		97.65	98.65	97.68	97.50
Allowed (%)	2.44		2.35	1.35	2.32	2.50
Disallowed (%)	0		0	0	0	0

Supplementary Information Table 2 | Yeast strains used in this study

Strain	Relevant Genotype	Source
BY4741	*MATa; his3Δ1; leu2Δ0; met15Δ0; ura3Δ0*	[45]
YJE608	*MATa; his3Δ1; leu2Δ0; met15Δ0; ura3Δ0; trp1Δ::LexA-ED-AD::* TRP1; ura3Δ0::$P_{minCYC1}$-NOG2-Myc-TEV-2xStrep::*URA3; TIF6-3xFLAG-3C-2xProtA::Hph*	This study
YJE735	*MATa; his3Δ1; leu2Δ0; met15Δ0; ura3Δ0; trp1Δ::LexA-ED-AD::* TRP1; ura3Δ0::$P_{minCYC1}$-NOG2-Myc-TEV-2xStrep::*URA3; TIF6-3xFLAG-3C-2xProtA::Hph;* $spb1^{D52A}$*::TEF-NrsR-T9*	This study
YJE743	*MATa his3Δ1 leu2Δ0 met15Δ0 ura3Δ0 trp1Δ::LexA-ED-AD::TRP1 SPB1::NrsR TEF-NrsR-T9*	This study
YJE744	*MATa his3Δ1 leu2Δ0 met15Δ0 ura3Δ0 trp1Δ::LexA-ED-AD::TRP1* $spb1^{D52A}$*::NrsR TEF-NrsR-T9*	This study
YJE869	*MATa his3Δ1 leu2Δ0 met15Δ0 ura3Δ0 trp1Δ::LexA-ED-AD::TRP1* $spb1^{D52AE769K}$*::NrsR TEF-NrsR-T9*	This study
YJE923	*MATa; his3Δ1; leu2Δ0; met15Δ0; ura3Δ0; trp1Δ::LexA-ED-AD::* TRP1; ura3Δ0::$P_{minCYC1}$-NOG2-Myc-TEV-2xStrep::*URA3; TIF6-3xFLAG-3C-2xProtA::HYG;* $spb1^{D52AE769K}$*::TEF-NrsR-T9*	This study
YJE1133	*MATa; his3Δ1; leu2Δ0; ura3Δ0; spb1Δ::HygR, pRS316-SPB1*	This study
YJE1221	*MATa; his3Δ1; leu2Δ0; ura3Δ0; spb1Δ::HygR, pRS313-SPB1*	This study
YJE1222	*MATa; his3Δ1; leu2Δ0; ura3Δ0; spb1Δ::HygR, pRS313-*$spb1^{D52A}$	This study
YJE1223	*MATa; his3Δ1; leu2Δ0; ura3Δ0; spb1Δ::HygR, pRS313-*$spb1^{D52A/E769K}$	This study
YJE1224	*MATa; his3Δ1; leu2Δ0; ura3Δ0; spb1Δ::HygR, pRS313-*$spb1^{D52A/K767N}$	This study
YJE1225	*MATa; his3Δ1; leu2Δ0; ura3Δ0; spb1Δ::HygR, pRS313-*$spb1^{E769K}$	This study
YJE1226	*MATa; his3Δ1; leu2Δ0; ura3Δ0; spb1Δ::HygR, pRS313-*$spb1^{K767N}$	This study

Supplementary Information Table 3 | Plasmids used in this study

Plasmid name	Description	Source
pRS246-GAL	Yeast 2 μ plasmid containing the GAL1 promoter (URA3)	Addgene
pJE649	pFA6a-3xFLAG-3C-6GLY-2xPrtA-HygMX	This study
pJE1047	pRS426-GAL-*NMD3*	This study
pJE1049	pRS426-GAL-*NOG2*	This study
pJE1051	pRS426-GAL-$nog2^{G369A}$	This study
pJE1069	pRS426-GAL-*SPB1-MTD-NoLS*	This study
pJE1071	pRS426-GAL-$spb1^{D52A}$-*MTD-NoLS*	This study
pJE1095	pRS316-*SPB1*	This study
pJE1105	pRS313-$spb1^{D52A/E769K}$	This study
pJE1106	pRS313-$spb1^{D52A/K767N}$	This study
pJE1107	pRS313-*SPB1*	This study
pJE1116	pRS313-$spb1^{E769K}$	This study
pJE1118	pRS313-$spb1^{K767N}$	This study
pJE1142	pRS313-$spb1^{D52A}$	This study
pRS316-uL18-GFP	Yeast CEN plasmid expressing uL18-GFP	[28]
pRS316-uS5-GFP	Yeast CEN plasmid expressing uS5-GFP	[28]

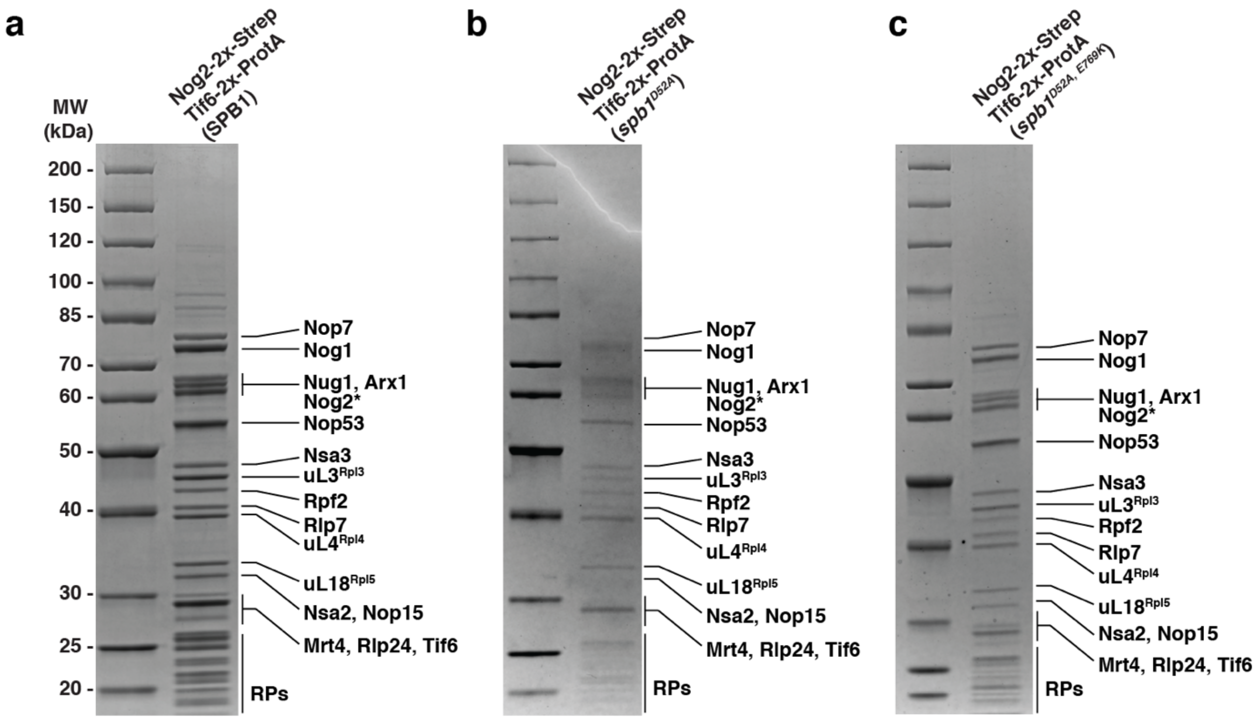

Supplementary Information Figure 1 | Purified Nog2·Tif6 pre-ribosomes used for cryo-EM analysis. **a**, Coomassie-stained 4 – 20% SDS-PAGE gel of the sample purified from the wildtype *SPB1* strain. **b**, Gel of sample derived from the *spb1*^{D52A} strain. **c**, Gel of sample derived from the *spb1*$^{D52A/E769K}$ strain. Protein identification was based on molecular weight, ambiguous bands were identified using mass spectrometry.

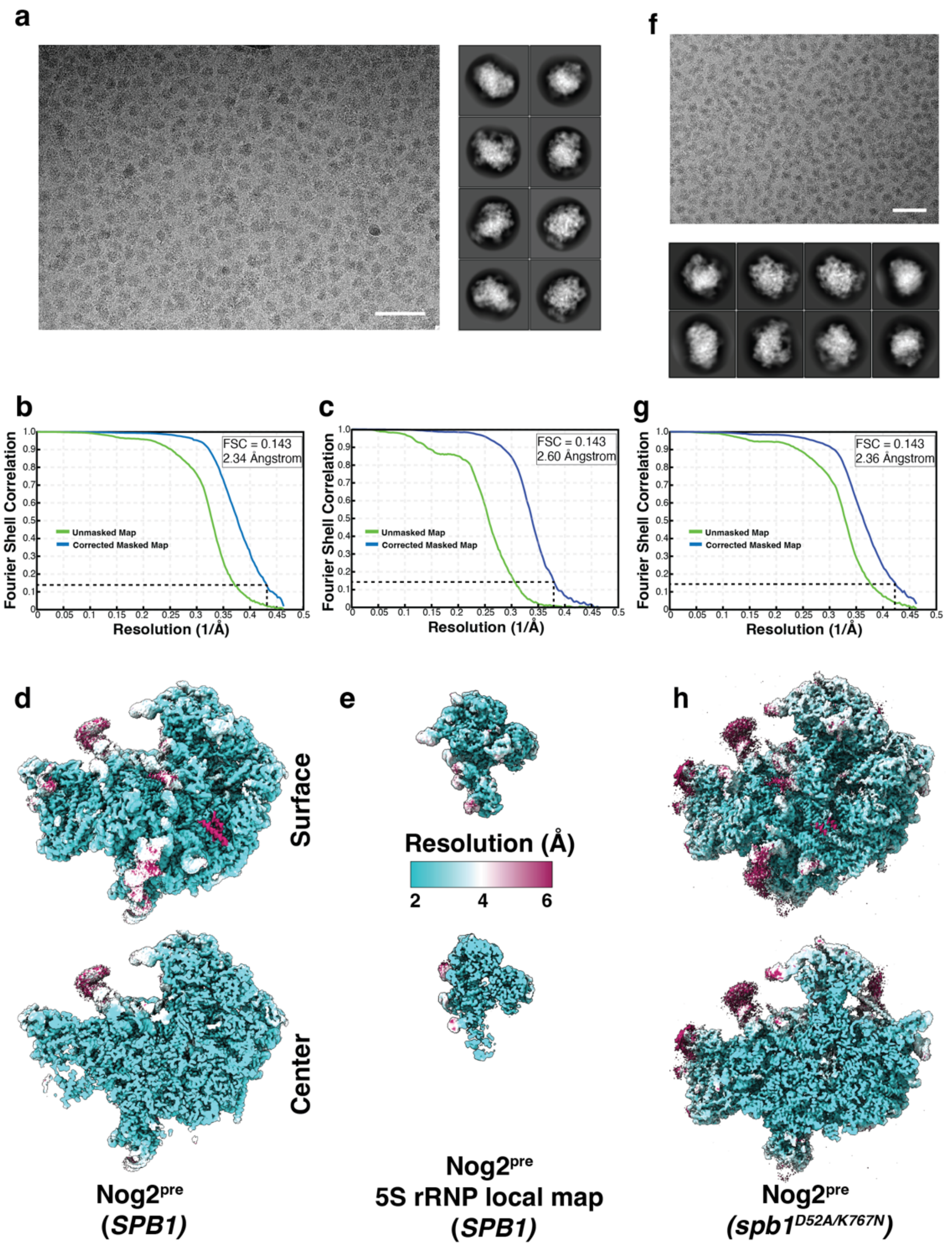

Supplementary Information Figure 2 | Micrographs, 2D classes, Fourier shell correlation (FSC) curves and ResMap plots of maps from *SPB1* (panels a-e) or *spb1*$^{D52A/E769K}$ (panels f-h) strains. **a**, Representative micrograph and 2D classes. Scale bar represents 50 µm. **b**, Fourier shell correlation of unmasked and masked maps with an overall resolution of 2.34 Å. **c**, Fourier shell correlation of unmasked and masked 5S rRNP local map with an overall resolution of 2.60 Å. **d**, Overall map (surface and sliced) colored according to local resolution estimates. **e**, Resolution distribution of local 5S RNP map. **f**, Representative micrograph and 2D classes. Scale bar represents 50 µm. **g**, Fourier shell correlation of unmasked and masked maps with an overall resolution of 2.36 Å. **h**, Overall pre-rotation map colored according to local resolution estimates.

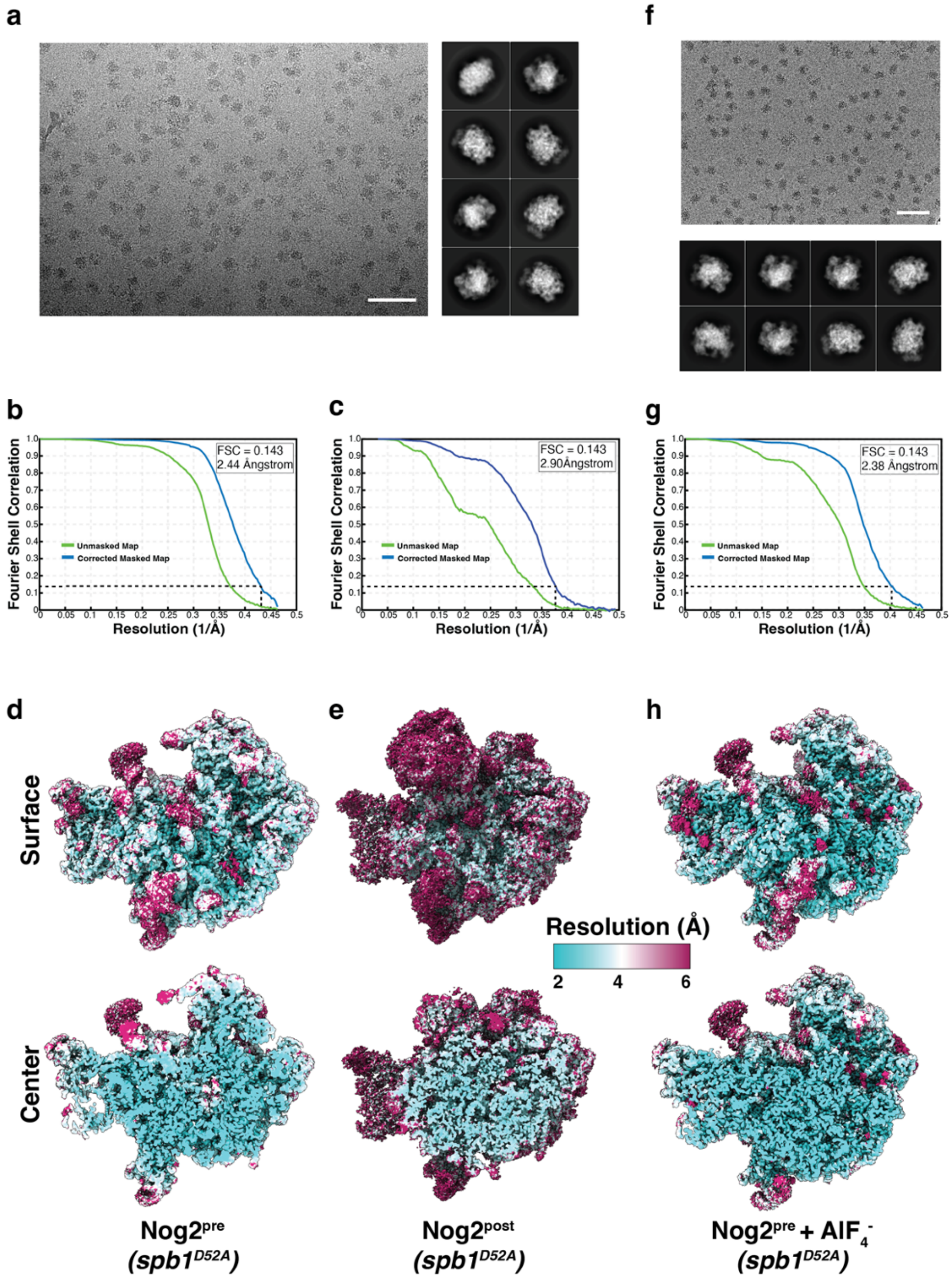

Supplementary Information Figure 3 | Micrographs, 2D classes, Fourier shell correlation (FSC) curves and ResMap plots of maps of Nog2^{pre} and Nog2^{post} (panels a-e) and Nog2^{pre}+ AlF_4- (panels f-h) purified from the *spb1^{D52A}* strain. **a**, Representative micrograph and 2D classes. Scale bar represents 50 µm. **b**, Fourier shell correlation of unmasked and masked Nog2^{pre} map with an overall resolution of 2.44 Å. **c**, Fourier shell correlation of unmasked and masked Nog2^{post} map with an overall resolution of 2.90 Å. **d**, Overall Nog2^{pre} map colored (surface and slice) according to local resolution estimates. **e**, Overall Nog2^{post} map is shown and colored according to local resolution estimates. **f**, Representative micrograph and 2D classes. Scale bar represents 50 µm. **g**, Fourier shell correlation of unmasked and masked Nog2^{pre} map with an overall resolution of 2.38 Å. **h**, Overall pre-rotation map colored according to the local resolution estimates

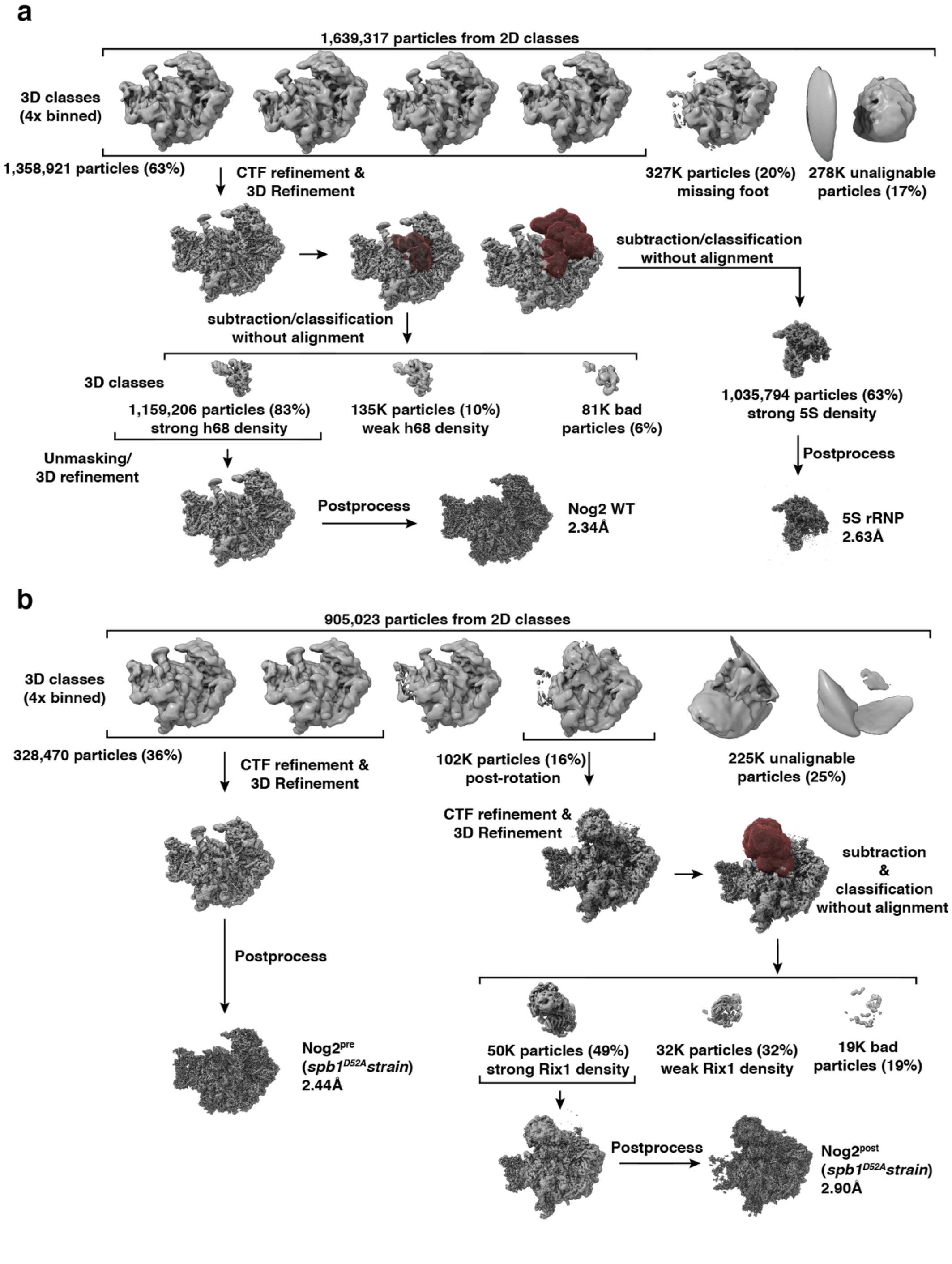

Supplementary Information Figure 4 | Cryo-EM data processing, 3D-classification and particle sorting scheme. a, Sorting and refinement scheme for Nog2^{pre} of the wild type *SPB1* sample. **b**, Sorting and refinement scheme for Nog2^{pre} and Nog2^{post} of the *spb1^{D52A}* sample. Map volumes are shown in gray, masks (dark red) used for subtraction and local classifications are shown aligned to their respective maps. Sorting and classification criteria are denoted, particle number and percentage of the total and final resolution are shown for each map.

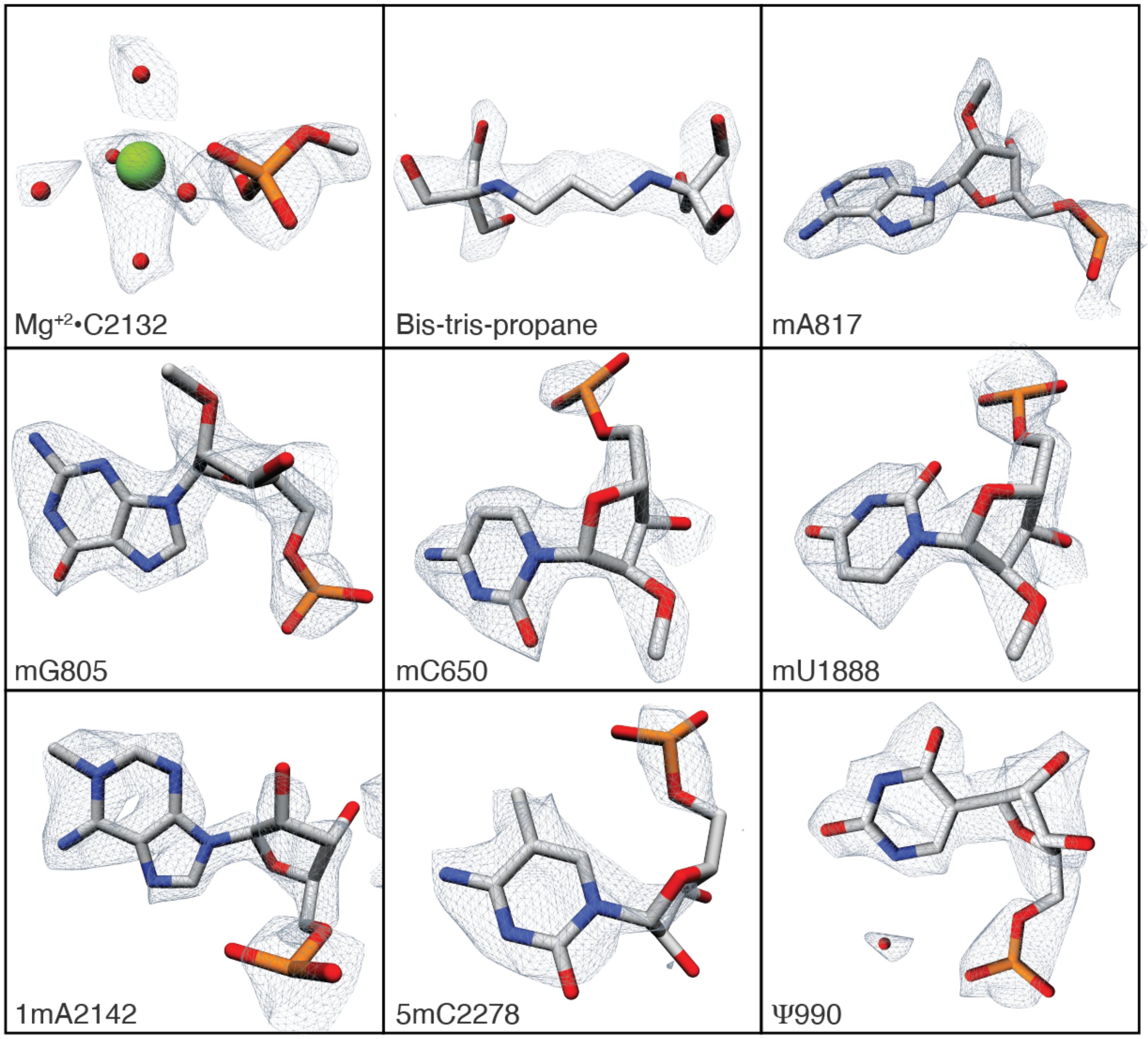

Supplementary Information Figure 5 | Representative Cryo-EM map densities for the wild-type Nog2^{pre} sample. Models and surrounding map density of coordinated magnesium ions, bis-tris-propane molecules and modified nucleotides identified within the Nog2^{pre} maps.

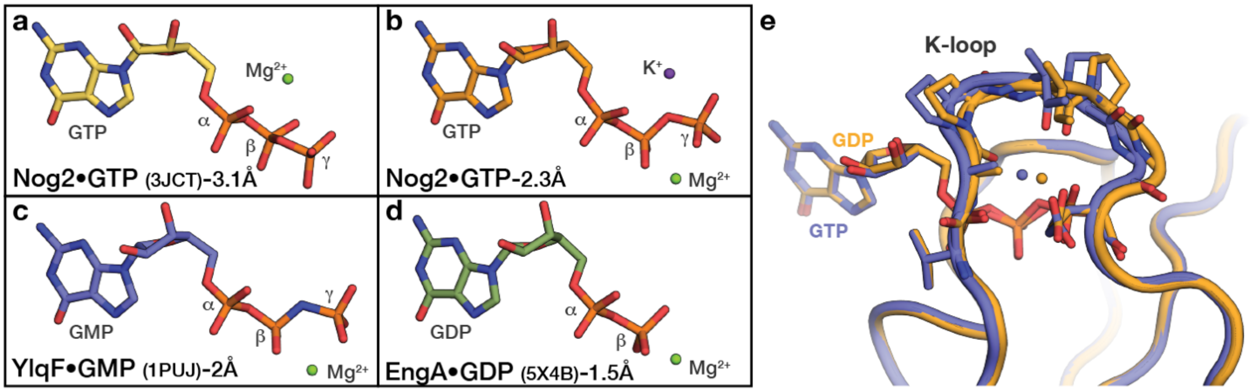

Extended Data Figure 6 | Map interpretation. Stick representations of nucleotides in selected K-loop GTPases. **a**, In the original Nog2[pre] structural model (PDB 3JCT), the γ-phosphate was placed into the density of the Mg^{2+} and the Mg^{2+} ion was placed in the density of the K^{+} ion. **b**, Our interpretation of the nucleotide density of Nog2 is consistent with high-resolution crystal structures (**c, d**) of the bacterial K-loop ATPases YlqF/RbgA (PDB 1PUJ) and EngA (PDB 5X4B) (**c**, **d**). **e**, Superposition of Nog2-GTP (blue) and Nog2-GDP (yellow) models shows that GTP hydrolysis causes a ~1Å shift in the position of the K-loop.

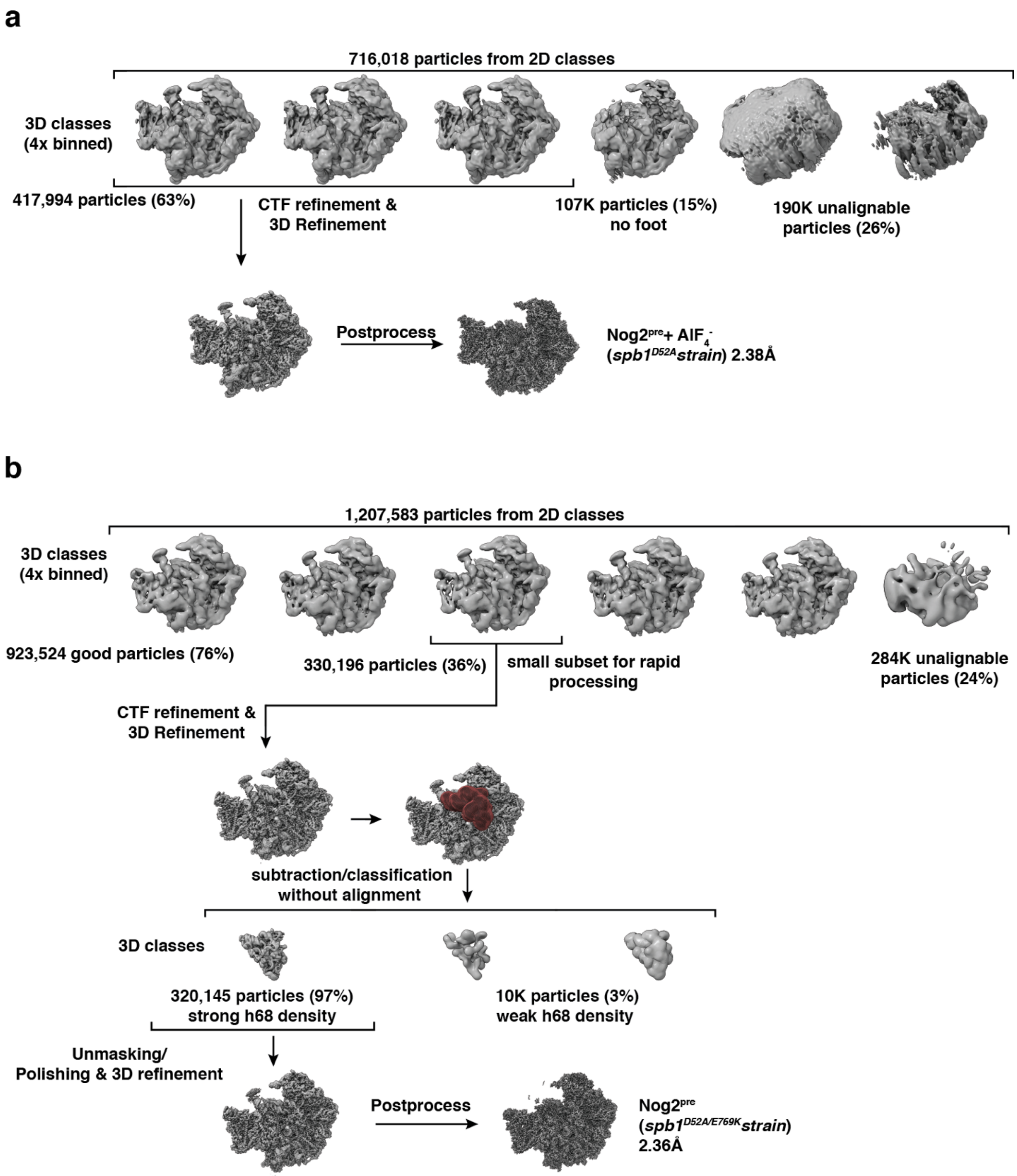

Supplementary Information Figure 7 | Cryo-EM data processing, 3D-classification and particle sorting scheme for Nog2·Tif6 intermediates. a, Sorting and refinement scheme for Nog2^{pre} purified from an *spb1*^{D52A} strain in which AlF_4- was added before grid preparation. **b,** Sorting and refinement scheme for Nog2^{pre} purified from an *spb1*$^{D52A/E769K}$ suppressor strain. Map volumes are shown in gray, masks (dark red) used for subtraction and local classifications are shown aligned to their respective maps. Sorting and classification criteria are denoted, particle number and percentage of the total and final resolution are shown for each map.

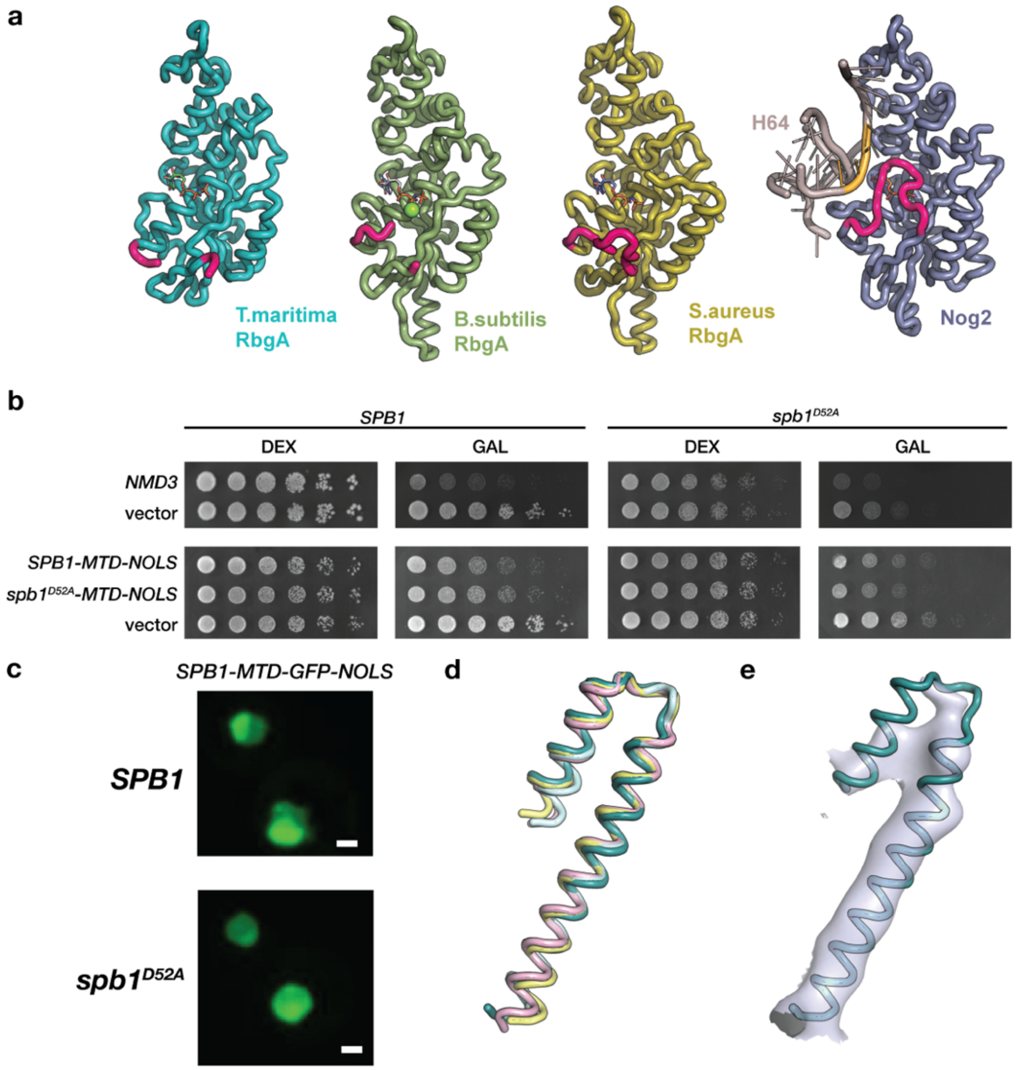

Supplementary Information Figure 8 | Overexpression of *NMD3* or *SPB1-MTD* does not rescue growth of *spb1*^{D52A} and location of *spb1*^{D52A} suppressor mutants in the C-terminal domain of Spb1. a, Cartoon representation of isolated bacterial Nog2 homologs (PDBs 3CNL, 1PUJ, and 6G12), showing that the K-loop (pink) is not present in a catalytically active conformation. When bound to the pre-60S, the K-loop of Nog2 is stabilized in its active conformation by interactions with H64 (far right). **b,** Overexpression of *NMD3* or *SPB1-MTD* does not rescue the slow growth phenotype of the *spb1*^{D52A} strain, instead showing a mild dominant negative effect. Plasmids containing Gal-inducible *NMD3, SPB1-MTD*, *spb1*^{D52A}*-MTD* or empty vector were transformed into the *SPB1* or *spb1*^{D52A} strains and plated on selective synthetic media containing Glucose (DEX) or Galactose (GAL). **c**, Light microscopy of yeast cells shows the proper nucleolar localization of overexpressed *SPB1-MTD-NOLS*. Images are pseudo-colored and scale bar represents 1 μm. **d**, Superposition of Alphafold models from *S.cerevisiae* (teal), *S.pombe* (pink), *D.melanogaster* (yellow) and *H.sapiens* (cyan) for the helical segment of Spb1 containing the suppressor mutations. **e**, *S.cerevisiae* model docked into the cryo-EM density from the NE1 (pdb 7U0H) pre-60S intermediate reconstruction[11].

References

45. Brachmann, C.B. et al. Designer deletion strains derived from Saccharomyces cerevisiae S288C: a useful set of strains and plasmids for PCR-mediated gene disruption and other applications. *Yeast* **14**, 115-32 (1998).
