# Supplementary material for: rRNA methylation by Spb1 regulates the GTPase activity of Nog2 during 60S ribosomal subunit assembly
Source: Nat Commun. 2023 Mar 2;14:1207. doi: 10.1038/s41467-023-36867-5 (PMC9981671; doi:10.1038/s41467-023-36867-5)
Supplement: Supplementary file 3 — Description of Additional Supplementary Files [file 41467_2023_36867_MOESM3_ESM.pdf]

File name: Supplementary Movie 1

Description: GTP, GDP-AlF4, GDP transitions in the active site of Nog2.
