# Supplementary material for: rRNA methylation by Spb1 regulates the GTPase activity of Nog2 during 60S ribosomal subunit assembly
Source: Nat Commun. 2023 Mar 2;14:1207. doi: 10.1038/s41467-023-36867-5 (PMC9981671; doi:10.1038/s41467-023-36867-5)
Supplement: Supplementary file 5 — Reporting Summary [file 41467_2023_36867_MOESM5_ESM.pdf]

nature portfolio

Corresponding author(s): Jan P. Erzberger

Last updated by author(s): Nov 21, 2022

Reporting Summary

Nature Portfolio wishes to improve the reproducibility of the work that we publish. This form provides structure for consistency and transparency in reporting. For further information on Nature Portfolio policies, see our Editorial Policies and the Editorial Policy Checklist.

Statistics

For all statistical analyses, confirm that the following items are present in the figure legend, table legend, main text, or Methods section.

n/a	Confirmed	
☒	☐	The exact sample size (*n*) for each experimental group/condition, given as a discrete number and unit of measurement
☐	☒	A statement on whether measurements were taken from distinct samples or whether the same sample was measured repeatedly
☒	☐	The statistical test(s) used AND whether they are one- or two-sided<br>*Only common tests should be described solely by name; describe more complex techniques in the Methods section.*
☒	☐	A description of all covariates tested
☒	☐	A description of any assumptions or corrections, such as tests of normality and adjustment for multiple comparisons
☐	☒	A full description of the statistical parameters including central tendency (e.g. means) or other basic estimates (e.g. regression coefficient) AND variation (e.g. standard deviation) or associated estimates of uncertainty (e.g. confidence intervals)
☒	☐	For null hypothesis testing, the test statistic (e.g. *F*, *t*, *r*) with confidence intervals, effect sizes, degrees of freedom and *P* value noted<br>*Give P values as exact values whenever suitable.*
☒	☐	For Bayesian analysis, information on the choice of priors and Markov chain Monte Carlo settings
☒	☐	For hierarchical and complex designs, identification of the appropriate level for tests and full reporting of outcomes
☒	☐	Estimates of effect sizes (e.g. Cohen's *d*, Pearson's *r*), indicating how they were calculated

Our web collection on statistics for biologists contains articles on many of the points above.

Software and code

Policy information about availability of computer code

Data collection	SerialEM 3.7.11
Data analysis	MotionCorr2, GCTF, RELION3.1 Beta, PHENIX v1.19.1, Coot v0.8.9.2, CHIMERA, Pymol v2.3.5, Proteome Discoverer 2.4

For manuscripts utilizing custom algorithms or software that are central to the research but not yet described in published literature, software must be made available to editors and reviewers. We strongly encourage code deposition in a community repository (e.g. GitHub). See the Nature Portfolio guidelines for submitting code & software for further information.

Data

Policy information about availability of data

All manuscripts must include a data availability statement. This statement should provide the following information, where applicable:

- Accession codes, unique identifiers, or web links for publicly available datasets
- A description of any restrictions on data availability
- For clinical datasets or third party data, please ensure that the statement adheres to our policy

The cryo-EM density maps and models have been deposited in EMDB and PDB with accession codes: EMD-26651/pdb-7UOO (Nog2pre from the SPB1 strain), EMD-26689 (Nog2pre 5S rRNP local map), EMD-26703/pdb-7UQZ (Nog2pre from spb1D52A strain), EMD-26799/pdb-7UUI (Nog2post from spb1D52A strain), EMD-26686/pdb-7UQB (Nog2pre with AlF4- from spb1D52A strain), EMD-26941/pdb-7V08 (Nog2pre from spb1D52A/E769K strain). Correspondence and requests for materials should be addressed to J.P.E.

Field-specific reporting

Please select the one below that is the best fit for your research. If you are not sure, read the appropriate sections before making your selection.

☒ Life sciences ☐ Behavioural & social sciences ☐ Ecological, evolutionary & environmental sciences

For a reference copy of the document with all sections, see nature.com/documents/nr-reporting-summary-flat.pdf

Life sciences study design

All studies must disclose on these points even when the disclosure is negative.

Sample size	The number of particles chosen for our cryo-EM reconstructions are similar or exceed the data set sizes of similar studies. No mathematical sample size calculation was performed. Detailed information about sample size, and technical or biological replicates are provided in Figure Legends or Extended Data.
Data exclusions	The particle selection strategy for our cryo-EM reconstructions are described in Extended Data, micrographs with poor Thon rings, devitrified ice, contamination from debris or extreme drift were discarded. For yeast growth experiments no relevant datasets have been excluded.
Replication	All genetic experiments were repeated at least three times with comparable results. All plasmid sequences and strains were confirmed by sequencing. Purification of pre-ribosomes was performed 2-5 times with comparable results.
Randomization	Particles are randomized and split into two datasets for calculation of Fourier Shell Correlation curves during cryo-EM resolution estimation in Relion 3.1. For purification of pre-ribosomes, genetic experiments comparing growth of different mutant strains and microscopy experiments randomization is not applicable.
Blinding	Purification of pre-ribosomes, grid preparation and data collection was performed by the same investigator using the same parameters for all samples. Yeast growth assays were also performed by the same investigator and did not require subjective interpretation of the results. All fluorescent microscopy was performed by the same investigator using similar parameters. Because no subjective interpretation of results was needed in this study blinding is not applicable.

Reporting for specific materials, systems and methods

We require information from authors about some types of materials, experimental systems and methods used in many studies. Here, indicate whether each material, system or method listed is relevant to your study. If you are not sure if a list item applies to your research, read the appropriate section before selecting a response.

Materials & experimental systems

n/a	Involved in the study	
☒	☐	Antibodies
☒	☐	Eukaryotic cell lines
☒	☐	Palaeontology and archaeology
☒	☐	Animals and other organisms
☒	☐	Human research participants
☒	☐	Clinical data
☒	☐	Dual use research of concern

Methods

n/a	Involved in the study	
☒	☐	ChIP-seq
☒	☐	Flow cytometry
☒	☐	MRI-based neuroimaging
